# Risk of testicular germ-cell tumours in relation to childhood physical activity

**DOI:** 10.1038/sj.bjc.6604109

**Published:** 2007-11-20

**Authors:** M B Cook, Y Zhang, B I Graubard, M V Rubertone, R L Erickson, K A McGlynn

**Affiliations:** 1 Division of Cancer Epidemiology and Genetics, National Cancer Institute, NIH, DHHS, Bethesda, MD 20892, USA; 2 Division of Environmental Health Science, Yale University School of Medicine, New Haven, CT 06510, USA; 3 US Army Center for Health Promotion and Preventive Medicine, Silver Spring, MD 21010, USA; 4 Walter Reed Army Institute of Research, Silver Spring, MD 20814, USA

**Keywords:** testicular cancer, physical activity, case – control

## Abstract

The US Servicemen's Testicular Tumor Environmental and Endocrine Determinants (STEED) case–control study of testicular germ-cell tumours (TGCTs) enrolled participants and their mothers in 2002–2005. Hours of sports or vigorous childhood physical activity per week were ascertained for three time periods; 1st–5th grades, 6th–8th grades and 9th–12th grades. Son- and mother-reports were analysed separately and included 539 control son–mother pairs and 499 case son–mother pairs. Odds ratios and 95% confidence intervals were produced. The analysis of the sons' responses found no relationship between childhood physical activity and TGCT, while the mothers' analysis found an inverse association, which was solely due to nonseminoma. Future studies should seek to validate responses further using recorded information sources such as school records.

Despite testicular germ-cell tumours (TGCTs) being the commonest malignancy in men aged 15–34 years of European ancestry (IARC, 2002; McGlynn *et al*, 2003), cryptorchidism, prior and family history of TGCT are the only consistently reported risk factors (McGlynn, 2001). Exposures early in life are thought to be integral in the initial stages of the disease process and in the 60% observed increased incidence between 1977 and 1997 (Purdue *et al*, 2005). Carcinoma *in situ* precedes TGCT and is postulated to arise from primordial germ cells (Skakkebaek *et al*, 1987), which may imply that *in utero* exposures are important (Moller, 1993). However, later exposures are still likely to influence risk, as has been indicated by the finding of a period effect in an analysis of incidence trends (Moller, 2001).

Increased levels of childhood physical activity have been reported to be protective against certain malignancies (Thune and Furberg, 2001). The US Servicemen's Testicular Tumor Environmental and Endocrine Determinants (STEED) Study was used to investigate whether childhood physical activity was associated with risk of TGCT and its two histologic subtypes of seminoma and nonseminoma.

## MATERIALS AND METHODS

Details of the US STEED Study have been published elsewhere (McGlynn *et al*, 2007). Briefly, between April 2002 and January 2005 participants aged 18–45 years with at least one serum sample stored in the Department of Defense Serum Repository (DoDSR, Silver Spring, MD, USA) were eligible for enrollment. Diagnoses of TGCT were limited to classic seminoma or nonseminoma (embryonal carcinoma, yolk sac carcinoma, choriocarcinoma, teratomas, mixed germ-cell tumour). The study was designed as a pair-matched, case–control study. Age (within 1 year), race (White, Black, other) and date of available serum sample (within 30 days) were the variables used for matching. In total, 767 cases and 928 controls were recruited, of which there were 720 matched case–control pairs. Permission was given by 1247 participants to contact their mothers. Forty-three mothers were found to be ineligible, 28 had their status pending and 16 could not be located. Of the 1160 eligible mothers contacted, 72 refused, giving a participation rate of 94%. Both members of a mother–son pair had to provide a response to the childhood physical activity question for a particular grade period if they were to be included in the respective analysis, as to allow for a direct comparison between mothers and sons. Overall there were 499 case son–mother pairs and 539 control son–mother pairs for analysis. The study was approved by the institutional review boards of the National Cancer Institute, Bethesda, MD, and the Walter Reed Army Institute for Research, Silver Spring, MD.

Participants were asked to report how many hours of sports or vigorous childhood physical activity they undertook during three time periods in childhood: 1st–5th grades, 6th–8th grades and 9th–12th grades. These time periods were chosen to correspond with ages in various schools (elementary school, middle school/junior high school, high school) with the hope of stimulating memory.

For a *post hoc* analysis, another question was used, which asked the son and mother to name the sports in which the son competed during 1st–12th grades. For this set of responses, the son's report was set as the ‘gold standard’ against which the mother's report was assessed. The mother's score was awarded two points for corroborating a sport named by the son, deducted half a point for failing to corroborate and deducted one point for providing a sport not specified by the son. Using the median of this score, the mothers' responses were divided into low- and high-agreement groups, both of which subsequently underwent re-analysis in an attempt to assess reporting accuracy.

### Statistical analysis

Odds ratios (ORs) and 95% confidence intervals were calculated to estimate the association of childhood physical activity with risk of TGCT using conditional logistic regression. To maximise the sample size in the analyses, unconditional logistic regression was also performed. As this involved breaking the match, risk estimates derived were first minimally adjusted, taking into account only the three matching factors. Further adjustment (in the fully adjusted model) was then made for the known TGCT risk factors: history of cryptorchidism and family history of testicular cancer. The results from all the analyses did not differ and therefore, only the fully adjusted estimates derived from the unconditional model are presented. When applicable, tests for linear trend in risk according to the medians of each quartile of a given ordered categorical variable were conducted to evaluate possible dose–response relationships. In addition, stratified analyses by tumour histology were performed to assess whether risks of seminoma and nonseminoma differed.

A Wald test was used to compute the *P*-values for testing the equivalence of the logarithm of the ORs between the mother's and son's reports of childhood physical activity (Korn and Graubard, 1999). The variances and covariances for this analysis were estimated from a leaving-one-out jack-knife method (Korn and Graubard, 1999). The Wald test was approximated by a *χ*^2^ distribution with a numerator degrees of freedom of three corresponding to the differences in the three ORs of the mothers and sons for the quartiles of childhood physical activity.

Kappa coefficients and intraclass correlation coefficients (Shrout and Fleiss, 1979) were generated to test for agreement between mothers and sons. In addition, kernel density plots for smoothing the histograms of the distribution of the difference in the reported hours of childhood physical activity per week for each time period were produced to examine potential reporting bias between sons and mothers. Statistical analyses were conducted with STATA 9.2 (StataCorp, College Station, TX, USA) and SAS 9.1 software (SAS Institute Inc, NC, USA). All tests were two sided, with *P*<0.05 defined as statistically significant.

## RESULTS

Characteristics of study participants are shown in Table 1. There were no significant differences in age and race, but cases were more likely to have a personal history of cryptorchidism (*P*=0.012) and a family history of testicular cancer (*P*=0.026). Participants were mainly White and TGCT incidence peaked at ages 20–24 years. Stratified by histology, the incidence peak for seminoma was slightly later than nonseminoma. Cryptorchidism was positively associated only with nonseminoma (*P*<0.001), while family history of testicular cancer was positively associated only with seminoma (*P*=0.001).

The risk estimates derived from logistic regression analyses of sons' reports are shown in Table 2. None of the ORs for all TGCT, seminoma or nonseminoma significantly deviated from the null hypothesis. Risk of seminoma appeared to be positively associated with childhood physical activity during 6th–8th grades, but none of the analyses met the statistically significant threshold of 0.05. If all cases (*n*=767) and controls (*n*=928) were included in the analysis of sons' data, regardless of whether their mother had returned a completed questionnaire, the estimates remained statistically nonsignificant. Also, although height has previously been reported to be positively associated with TGCT risk in this study (McGlynn *et al*, 2007), it was not associated with childhood physical activity (all *P*>0.05) and was, therefore, not included in the fully adjusted model.

The risk estimates calculated from mothers' responses (Table 3) were markedly different from the sons' analysis. Increasing levels of reported childhood physical activity were inversely associated with TGCT risk. In each time period analysed, at least one of the three levels of activity was significantly associated with decreased risk particularly during early grades, as were all of the *P*-values for linear trend. Childhood physical activity in the highest grades (9th–12th grades) was only associated with decreased risk at the highest level of exertion (⩾21 h per week: OR=0.60, 95% CI: 0.42–0.87).

The observed protective effect of increased childhood physical activity was solely and consistently associated with nonseminoma, all the risk estimates being lower relative to the TGCT analysis, and the *P*-values for linear trend also lower. None of the ORs for seminoma were significant.

The differences between sons' and mothers' responses were more pronounced at the younger ages: the ORs were statistically significant for grades 1st–5th (*χ*_3_^2^=14.02, *P*=0.003) and 6th–8th (*χ*_3_^2^=12.83, *P*=0.005), but not for grades 9th–12th (*χ*_3_^2^=6.33, *P*=0.097). When stratified by histologic group the *χ*^2^ estimates became statistically nonsignificant for the differences within seminoma analyses (1st–5th, *χ*_3_^2^=1.96, *P*=0.58; 6th–8th, *χ*_3_^2^=4.39, *P*=0.22; 9th–12th, *χ*_3_^2^=0.59, *P*=0.90), while the nonseminoma comparisons were statistically significant for the first two time periods (1st–5th, *χ*_3_^2^=6.08, *P*=0.0003; 6th–8th, *χ*_3_^2^=4.03, *P*=0.007; 9th–12th, *χ*_3_^2^=2.18, *P*=0.087).

Kernel density plots of the difference between sons' and mothers' responses to questions of childhood physical activity were produced to examine potential reporting bias. These plots were stratified by histologic group and time period analysed. The nonseminoma distributions appeared slightly more positive relative to the control and seminoma groups. This observation shows the extent of the discrepancy of childhood physical activity levels reported by the mother and son; mothers reported lower levels of childhood physical activity if their son had been diagnosed with nonseminoma.

Linear regression showed that the difference between sons' and mothers' responses increased with the mothers' total number of live male births, but not parity *per se*, and decreased with the mothers' level of education. However, the *r*^2^ values were very low, indicative of the large variation present.

Agreement between sons' and mothers' responses categorised into quartiles was low, with 28% agreement for 1st–5th grades, 32% for 6th–8th and 32% for 9th–12th, with corresponding kappa coefficients 0.02, 0.08 and 0.08. Using the continuous data, intraclass correlation coefficients were 0.13 for 1st–5th grades, 0.13 for 6th–8th and 0.19 for 9th–12th. The low levels of agreement prompted a *post hoc* subgroup analysis in which an attempt was made to stratify mothers' response based on the accuracy of recall, as described in Materials and Methods. The mothers' responses were divided into low- and high-agreement groups. The ORs for these groups are shown in Table 4, the point estimates being very similar.

## DISCUSSION

This investigation has analysed the relationship between childhood physical activity and TGCT risk using data from the US STEED Study.

An examination of the sons' responses found no significant associations, although the point estimates for seminoma during 6th–8th grades did approach significance and may indicate that childhood physical activity increases risk of seminoma (Table 2). Importantly, the sons' data gave no suggestion of any protective effect of increased childhood physical activity on TGCT, whereas the mothers' responses pointed to a consistent and histologically specific inverse association. Increased childhood physical activity, during all three time periods analysed, was inversely associated with TGCT risk (Table 3). When stratified by histologic group, it appeared that the association was restricted to nonseminoma. This observation is unexpected, especially given that, even at later grades, the analysis of sons' data provided no indication of a protective association. Whether mothers' or sons' responses should be more reliable is unclear.

In an attempt to test the possibility of poor recall in the mothers, a *post hoc* analysis that stratified the mothers' responses on their recall accuracy was conducted. Recall accuracy was estimated by determining whether the mother could name the sports her son played, as detailed by the son. The rationale for this comparison was that it was likely that the son could accurately recall the particular sports he played. The results of the analysis, which compared low- and high-agreement groups, did not dramatically differ (Table 4). In the nonseminoma analysis, the levels of statistical significance were somewhat attenuated in the high-agreement group, but the differences were too slight to reject the results of the main analysis, especially given that the *post hoc* analysis was based on smaller numbers due to stratification.

The findings raise the question: does childhood physical activity protect against TGCT (particularly nonseminoma) or not? The mothers' results would be easier to dismiss if the effect was not so consistent across time periods and, more importantly, within one histologic subgroup. The latter observation gives credence to the results; if the protective effect on TGCT was caused by bias or chance, one may expect the effect to be equal when stratified by histology. Given parental nurturing responsibilities, it is conceivable that differential misclassification could have been stronger in mothers compared with sons, but it is both unlikely and nonsensical that the bias would be associated with nonseminoma *per se*. Nonseminoma is a more clinically aggressive tumour that occurs at a slightly younger age than seminoma (Gori *et al*, 2005), however, as both histologic types occur in adults, it seems unlikely that misclassification or rumination bias affected the mothers' responses. Conversely, the role of chance cannot be ruled out, especially since the sons' analyses support the null hypothesis of no association between physical activity and TGCT risk.

Two previous case–control studies have found physical activity to be protective against TGCT (UK Testicular Cancer Study Group, 1994; Gallagher *et al*, 1995). The first of these is the United Kingdom Testicular Cancer Study (UK Testicular Cancer Study Group, 1994). This study comprised 794 TGCT cases diagnosed between 1984 and 1987 and found that participating in various sports at age 16 years, age 20 years or 1 year prior to diagnosis was protective against development of TGCT (UK Testicular Cancer Study Group, 1994). A second study, conducted in Canada, also found an inverse relationship between recreational activity and TGCT (Gallagher *et al*, 1995). However, neither of these previous case–control studies stratified their analyses by histology or reported childhood physical activity data collected from mothers. Three studies have found no association between physical activity and testis cancer (Paffenbarger *et al*, 1987; Dosemeci *et al*, 1993; Thune and Lund, 1994). None of these three studies distinguished between germ and nongerm tumour types and it is thus conceivable that any true effect on TGCT risk has been masked. Further, two of these studies had fewer than 50 cases (Paffenbarger *et al*, 1987; Thune and Lund, 1994) while the third study based physical activity purely on adult occupational history and is, therefore, likely to have missed the important time-window of exposure for TGCT risk (Dosemeci *et al*, 1993). Only one previous study has inferred that physical activity and TGCT risk are positively associated (Srivastava and Kreiger, 2000). Analysing data from 212 cases, the study found that moderate and strenuous recreational activity levels during the mid-teens approximately doubled the risk of TGCT. However, the sum of the evidence would suggest that increased physical activity is protective against TGCTs, thus the results of the mothers' analysis may warrant further consideration.

For testicular congenital abnormalities, mother's responses were used to verify or supplement self-report data (Stang *et al*, 2001). While concerns were raised over the combination of biases, for factual objective variables this methodology is likely to be the most appropriate. Combining mother and son responses for subjective, quantitative variables, where a larger margin of error may be expected, would appear not to be endorsed by our results.

This study has the strengths of large numbers, interviewer-conducted data collection, and inquiries tied to specific periods (i.e. grades in school). The main limitation is the lack of validation of the responses of sons and mothers.

Our analysis of mothers' data suggests that increased childhood physical activity may decrease the risk of nonseminoma, although the sons' data do not support this. Studies with validated responses against other sources of information, such as school records, are needed to confirm or refute our findings.

## Figures and Tables

**Table 1:** Characteristics of study participants, STEED Study, 2002–2005

	**Controls (*n*=539)**		**TGCTs (*n*=499)**			**Seminoma (*n*=199)**			**Nonseminoma (*n*=300)**		
**Variable**	**No.**	**%**	**No.**	**%**	***P*-value^a^**	**No.**	**%**	***P*-value^a^**	**No.**	**%**	***P*-value^a^**
*Age (years)*											
<20	21	3.9	26	5.2		4	2.0		22	7.3	
20–24	193	35.8	169	33.9		40	20.1		129	43.0	
25–29	167	31.0	157	31.5		71	35.7		86	28.7	
30–34	91	16.9	77	15.4		40	20.1		37	12.3	
35–39	51	9.5	54	10.8		34	17.1		20	6.7	
⩾40	16	3.0	16	3.2	0.83	10	5.0	<0.001	6	2.0	0.029
											
*Race*											
White	486	90.2	441	88.4		170	85.4		271	90.3	
Black	13	2.4	11	2.2		4	2.0		7	2.3	
Other	40	7.4	47	9.4	0.50	25	12.6	0.090	22	7.3	1.00
											
*Cryptorchidism*											
No	527	97.8	473	94.8		194	97.5		279	93.0	
Yes	12	2.2	26	5.2	0.012	5	2.5	0.81	21	7.0	<0.001
											
*Family history of testicular cancer* b											
No	529	98.1	478	95.8		186	93.5		292	97.3	
Yes	10	1.9	21	4.2	0.026	13	6.5	0.0012	8	2.7	0.44

STEED=Servicemen's Testicular Tumor Environmental and Endocrine Determinants; TGCT=testicular germ-cell tumour.

a Based on *χ*^2^ analysis.

b Family history among first- and second-degree relatives.

**Table 2:** An analysis of childhood physical activity on TGCT risk using sons' reports in the STEED Study, 2002–2005

	**Controls**	**TGCT**			**Seminoma**			**Nonseminoma**		
**Hours per week in sports or vigorous physical activity**	**No.**	**No.**	**Odds ratio^a^**	**95% confidence**	**No.**	**Odds ratio^a^**	**95% confidence**	**No.**	**Odds ratio^a^**	**95% confidence**
*1st*–*5th grades*^b^										
⩽5	178	146	1.00	Referent	57	1.00	Referent	89	1.00	Referent
6–10	157	156	1.21	0.88, 1.66	65	1.30	0.85, 2.00	91	1.12	0.77,1.62
11–15	74	57	0.93	0.61, 1.40	22	1.02	0.58, 1.82	35	0.88	0.54,1.42
⩾16	112	120	1.32	0.93, 1.85	45	1.23	0.76, 1.97	75	1.36	0.92,2.03
*P for trend*			*0.22*			*0.55*			*0.20*	
										
*6th*–*8th grades*^c^										
⩽7	148	120	1.00	Referent	38	1.00	Referent	82	1.00	Referent
8–12	155	143	1.09	0.78, 1.52	63	1.56	0.97, 2.51	80	0.87	0.59,1.29
13–20	135	137	1.19	0.84, 1.67	60	1.56	0.96, 2.53	77	0.99	0.66,1.47
⩾21	90	89	1.21	0.82, 1.78	33	1.41	0.81, 2.45	56	1.14	0.74,1.77
*P for trend*			*0.29*			*0.33*			*0.42*	
										
*9th*–*12th grades*^d^										
⩽8	129	113	1.00	Referent	44	1.00	Referent	69	1.00	Referent
9–15	175	164	1.02	0.73, 1.43	61	1.02	0.64, 1.62	103	1.05	0.71,1.55
16–20	98	91	1.06	0.72, 1.56	45	1.42	0.85, 2.36	46	0.85	0.53,1.35
⩾21	114	113	1.07	0.74, 1.55	44	1.05	0.63, 1.73	69	1.07	0.69,1.64
*P for trend*			*0.67*			*0.60*			*0.98*	

STEED=Servicemen's Testicular Tumor Environmental and Endocrine Determinants; TGCT=testicular germ-cell tumour.

a Logistic regression adjusted for reference age, race, serum date, cryptorchidism and family history of testicular cancer.

b Excludes individuals with data unknown (40 controls, 36 cases).

c Excludes individuals with data unknown (33 controls, 26 cases).

d Excludes individuals with data unknown (45 controls, 34 cases).

**Table 3:** An analysis of childhood physical activity on TGCT risk using mothers' reports in the STEED Study, 2002–2005

	**Controls**	**TGCT**			**Seminoma**			**Nonseminoma**		
**Hours per week in sports or vigorous physical activity**	**No.**	**No.**	**Odds ratio^a^**	**95% confidence**	**No.**	**Odds ratio^a^**	**95% confidence**	**No.**	**Odds ratio^a^**	**95% confidence**
*1st*–*5th grades*^b^										
⩽5	124	159	1.00	Referent	47	1.00	Referent	112	1.00	Referent
6–10	142	130	0.70^*^	0.50, 0.98	57	1.18	0.74, 1.90	73	0.52^**^	0.35, 0.77
11–15	121	90	0.59^**^	0.41, 0.85	49	1.16	0.71, 1.89	41	0.39^***^	0.25, 0.60
⩾16	134	100	0.56^**^	0.39, 0.80	36	0.81	0.49, 1.36	64	0.47^***^	0.32, 0.71
*P for trend*			*0.001*			*0.40*			<*0.001*	
										
*6th*–*8th grades*^c^										
⩽7	115	150	1.00	Referent	48	1.00	Referent	102	1.00	Referent
8–12	139	115	0.60^**^	0.42, 0.85	48	0.84	0.52, 1.37	67	0.50^**^	0.33, 0.75
13–20	169	151	0.68^*^	0.49, 0.95	68	1.01	0.64, 1.60	83	0.54^**^	0.37, 0.79
⩾21	105	73	0.52^**^	0.35, 0.77	30	0.79	0.46, 1.35	43	0.43^***^	0.27, 0.68
*P for trend*			*0.003*			*0.54*			<*0.001*	
										
*9th*–*12th grades*^d^										
⩽8	128	152	1.00	Referent	55	1.00	Referent	97	1.00	Referent
9–15	184	162	0.74	0.54, 1.02	68	0.88	0.57, 1.37	94	0.68^*^	0.47, 0.98
16–20	78	77	0.85	0.57, 1.26	35	1.19	0.70, 2.02	42	0.72	0.45, 1.15
⩾21	126	90	0.60^**^	0.42, 0.87	36	0.82	0.50, 1.37	54	0.53**	0.35, 0.81
*P for trend*			*0.018*			*0.72*			*0.005*	

STEED=Servicemen's Testicular Tumor Environmental and Endocrine Determinants; TGCT, testicular germ-cell tumour.

* *P*<0.05.

** *P*<0.01.

*** *P*<0.001.

a Logistic regression adjusted for reference age, race, serum date, cryptorchidism and family history of testicular cancer.

b Excludes individuals with data unknown (40 controls, 36 cases).

c Excludes individuals with data unknown (33 controls, 26 cases).

d Excludes individuals with data unknown (45 controls, 34 cases).

**Table 4:** A subgroup analysis of childhood physical activity on TGCT risk using mothers' reports with low and high-agreement scores in the STEED Study, 2002–2005

	**TGCT**		**Seminoma**		**Nonseminoma**	
	**Agreement^a^**		**Agreement^a^**		**Agreement^a^**	
**Hours per week in sports or vigorous physical activity**	**Low**	**High**	**Low**	**High**	**Low**	**High**
*1st*–*5th grade*						
⩽5	1.00	1.00	1.00	1.00	1.00	1.00
6–10	0.77	0.62	1.90	0.75	0.43^**^	0.57^*^
11–15	0.57^*^	0.59	1.07	1.16	0.36^**^	0.39^**^
⩾16	0.58^*^	0.53^*^	0.97	0.71	0.46^**^	0.47^*^
*P for trend*	*0.016*	*0.028*	*0.51*	*0.68*	*0.004*	*0.010*
						
*6th*–*8th grade*						
⩽7	1.00	1.00	1.00	1.00	1.00	1.00
8–12	0.56^*^	0.63	1.09	0.62	0.34^**^	0.66
13–20	0.62^*^	0.76	0.94	1.10	0.48^**^	0.62
⩾21	0.48^*^	0.56^*^	0.89	0.69	0.33^**^	0.54
*P for trend*	*0.010*	*0.094*	*0.69*	*0.71*	*0.001*	*0.07*8
						
*9th*–*12th grade*						
⩽8	1.00	1.00	1.00	1.00	1.00	1.00
9–15	0.78	0.69	0.98	0.86	0.69	0.64
16–20	0.95	0.79	1.37	1.23	0.76	0.67
⩾21	0.49^**^	0.69	0.66	1.04	0.41^**^	0.61
*P for trend*	*0.021*	*0.32*	*0.42*	*0.63*	*0.011*	*0.19*

STEED=Servicemen's Testicular Tumor Environmental and Endocrine Determinants; TGCT, testicular germ-cell tumour.

* *P*<0.05

** *P*<0.01

a Agreement score was based on a questionnaire, which asked respondents to specify sports the son played between 1st–12th grades.
